# Forelimb-hindlimb developmental timing changes across tetrapod phylogeny

**DOI:** 10.1186/1471-2148-7-182

**Published:** 2007-10-01

**Authors:** Olaf RP Bininda-Emonds, Jonathan E Jeffery, Marcelo R Sánchez-Villagra, James Hanken, Matthew Colbert, Claude Pieau, Lynne Selwood, Carel ten Cate, Albert Raynaud, Casmile K Osabutey, Michael K Richardson

**Affiliations:** 1 Institute of Biology, University of Leiden, Kaiserstraat 63, 2311GP, Leiden, The Netherlands; 2 Institut für Spezielle Zoologie und Evolutionsbiologie mit Phyletischem Museum, Friedrich-Schiller-Universität Jena, Erbertstrasse 1, 07743 Jena, Germany; 3 Palaeontologisches Institut und Museum, Karl Schmid-Strasse 4, CH-8006 Zürich, Switzerland; 4 Museum of Comparative Zoology, Harvard University, 26 Oxford Street, Cambridge, MA 02138, USA; 5 Department of Geological Sciences, The University of Texas, Austin, TX 78712, USA; 6 Laboratoire de Biochimie du Développement, Institut Jacques Monod, CNRS et Université Paris 6 et 7, Tour 43-33, E3, 2, place Jussieu, 75251 Paris Cedex 05, France; 7 Department of Zoology, Gate 12, University of Melbourne, Victoria. 3010, Australia; 8 Formerly of Laboratoire Pasteur (Embryologie expérimentale), 20 rue des Moulins, 95110 Sannois, France; 9 Department of Anatomy, St George's Hospital Medical School, Tooting, London SW17 0RE, UK

## Abstract

**Background:**

Tetrapods exhibit great diversity in limb structures among species and also between forelimbs and hindlimbs within species, diversity which frequently correlates with locomotor modes and life history. We aim to examine the potential relation of changes in developmental timing (heterochrony) to the origin of limb morphological diversity in an explicit comparative and quantitative framework. In particular, we studied the relative time sequence of development of the forelimbs versus the hindlimbs in 138 embryos of 14 tetrapod species spanning a diverse taxonomic, ecomorphological and life-history breadth. Whole-mounts and histological sections were used to code the appearance of 10 developmental events comprising landmarks of development from the early bud stage to late chondrogenesis in the forelimb and the corresponding serial homologues in the hindlimb.

**Results:**

An overall pattern of change across tetrapods can be discerned and appears to be relatively clade-specific. In the primitive condition, as seen in Chondrichthyes and Osteichthyes, the forelimb/pectoral fin develops earlier than the hindlimb/pelvic fin. This pattern is either retained or re-evolved in eulipotyphlan insectivores (= shrews, moles, hedgehogs, and solenodons) and taken to its extreme in marsupials. Although exceptions are known, the two anurans we examined reversed the pattern and displayed a significant advance in hindlimb development. All other species examined, including a bat with its greatly enlarged forelimbs modified as wings in the adult, showed near synchrony in the development of the fore and hindlimbs.

**Conclusion:**

Major heterochronic changes in early limb development and chondrogenesis were absent within major clades except Lissamphibia, and their presence across vertebrate phylogeny are not easily correlated with adaptive phenomena related to morphological differences in the adult fore- and hindlimbs. The apparently conservative nature of this trait means that changes in chondrogenetic patterns may serve as useful phylogenetic characters at higher taxonomic levels in tetrapods. Our results highlight the more important role generally played by allometric heterochrony in this instance to shape adult morphology.

## Background

What evolutionary mechanisms are responsible for differences in the relative size of body parts among organisms? This basic question has long been confronted by biologists, for example, by J. S. Huxley, in his *Problems of Relative Growth *[1]. When considering the tetrapod limb, one might ask why the forelimbs are relatively larger or smaller than the hindlimbs in some species, and how these differences have arisen during evolution.

Tetrapods exhibit great diversity in limb structures among species and in differences between fore- and hindlimbs within species, which typically are correlated with locomotor modes and life history [2]. Among mammals, the relatively large wings of an adult bat exhibit a striking contrast in size and proportions to its legs, whereas the disparity in most other living eutherians (e.g. mice) is more modest. Kangaroos represent the opposite situation, having relatively massive hindlimbs. These differences are not restricted to mammals but characterize tetrapods as a whole, as evidenced when considering a bird or a frog or a turtle, or fossils such as *Tyranosaurus rex*, which has huge hindlimbs and tiny forelimbs.

A largely unanswered question is how these differences are reflected in the ontogenetic development of the limbs. Limbs are one of the best studied systems in both evolution and development [3] and display a sequence of well-defined temporal events, such as formation of the apical ectodermal ridge (AER) and the chondrification of skeletal elements. We examine here the extent to which features of early limb development, especially chondrogenesis, might be associated with obvious differences in forelimb and hindlimb size or function in the adult. We concentrate on heterochrony, the evolutionary change in developmental timing, a process which is thought to be important and common in evolution [4]. In particular, we examine the relative timing of developmental events during ontogeny across the phylogeny of the species examined (sequence heterochrony; sensu [5]).

## Results

**Figure 1:**
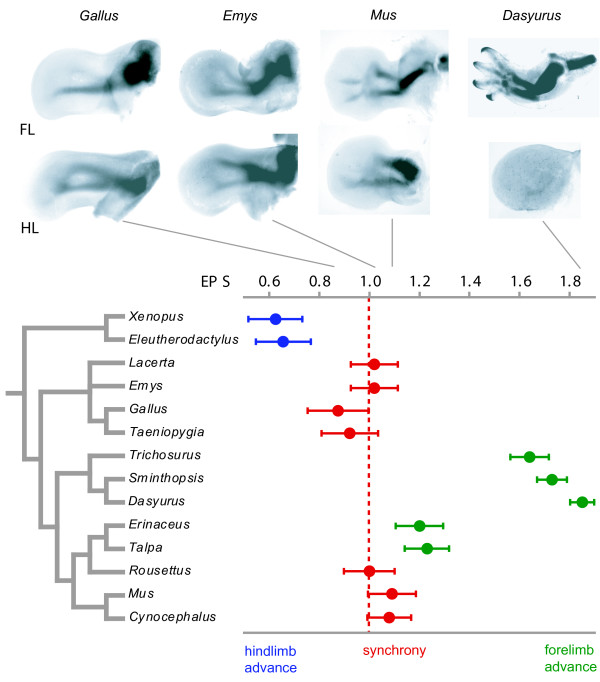
Average event-pair scores (EPSs) plotted on a phylogeny of the taxa examined, with visual examples of forelimb (FL) and hindlimb (HL) across the tree. Plot symbols in blue and green are significantly less than and greater than one, respectively; forelimb-hindlimb synchrony, in red. The tree is derived from references 33 and 34.

The average event-pair score (EPS; see Methods) was plotted for each species (Table 1, Fig. 1). The two anurans (*Xenopus*, *Eleutherodactylus*) show average EPS scores significantly less than one, indicating that hindlimb development generally precedes that of the forelimb (noted by [6,7]). The two birds (*Taeniopygia*, *Gallus*) also tend to show an advance in hindlimb development – small differences between fore- and hindlimb timing can be detected visually (for example, in the figures of *Gallus *in [8]) – but are not significantly different from forelimb-hindlimb synchrony together with the remaining diapsids *Lacerta *and *Emys*. Among mammals, the marsupials (*Trichosurus*, *Dasyurus *and *Sminthopsis*) and eulipotyphlan insectivores (*Erinaceus *and *Talpa*) all show significant advances in forelimb development. The generally smaller forelimb advances among the remaining eutherian mammals were not significantly different from synchronous development.

**Table 1:** Statistics on the temporal distribution of developmental events

**Species (common name)**	***N***	**Avg. Stage**	***EPS***	**Student's *t***
*Eleutherodactylus coqui *(tree frog)	**12**	3.091	0.656 ± 0.110	*t*_63 _= -3.136 (*P *= 0.0026)
*Xenopus laevis *(clawed toad)	**6**	4.860	0.625 ± 0.108	*t*_63 _= -3.473 (*P *= 0.0009)
*Emys orbicularis *(pond turtle)	**10**	3.333	1.020 ± 0.093	*t*_99 _= 0.215 (*P *= 0.8305)
*Lacerta viridis *(wall lizard)	**12**	2.857	1.020 ± 0.093	*t*_99 _= 0.215 (*P *= 0.8305)
*Gallus gallus *(chicken)	**10**	2.188	0.875 ± 0.121	*t*_63 _= -1.033 (*P *= 0.3054)
*Taeniopygia guttata *(zebra finch)	**7**	3.091	0.922 ± 0.112	*t*_63 _= -0.697 (*P *= 0.4882)
*Trichosurus vulpecula *(brushtail possum)	**15**	2.500	1.640 ± 0.076	*t*_99 _= 8.432 (*P *< 0.0001)
*Sminthopsis macroura *(stripe-faced dunnart)	**5**	8.000	1.730 ± 0.060	*t*_99 _= 12.155 (*P *< 0.0001)
*Dasyurus viverrinus *(marsupial cat)	**11**	3.077	1.850 ± 0.048	*t*_99 _= 17.732 (*P *< 0.0001)
*Erinaceus europaeus *(hedgehog)	**10**	3.333	1.200 ± 0.094	*t*_99 _= 2.121 (*P *= 0.0364)
*Talpa europea *(mole)	**10**	3.333	1.230 ± 0.089	*t*_99 _= 2.596 (*P *= 0.0109)
*Mus musculus *(mouse)	**12**	2.857	1.090 ± 0.096	*t*_99 _= 0.933 (*P *= 0.3533)
*Rousettus amplexicaudatus *(fruitbat)	**10**	3.800	1.000 ± 0.101	*t*_89 _= 0.000 (*P *= 1.000)
*Cynocephalus variegatus *(flying lemur)	**8**	3.333	1.080 ± 0.088	*t*_99 _= 0.905 (*P *= 0.3677)

The event-pair score (EPS; presented as average ± SE) may range from 2 (forelimb advanced over hindlimb) to 0 (hindlimb advanced over forelimb). Two-tailed Student's *t*-tests were used to determine if the EPS was significantly different from 1. *N *is the number of stages of limb development identified and Avg. Stage is the mean number of events that occurred at each stage.

An analysis of variance in combination with Fisher's PLSD test detected four major clades among the tetrapod species we examined (*F*_3,1242 _= 66.418, *P *< 0.0001):

1. anuran amphibians: strong, significant hindlimb acceleration with an average EPS of 0.641 ± 0.076 (SE);

2. diapsids (lizards, turtles, and birds): slight, but insignificant hindlimb acceleration with an average EPS of 0.973 ± 0.051;

3. eutherian mammals: slight, but insignificant forelimb acceleration with an average EPS of 1.122 ± 0.042;

4. marsupial mammals: strong, significant forelimb acceleration with an average EPS of 1.740 ± 0.036.

## Discussion

**Figure 2:**
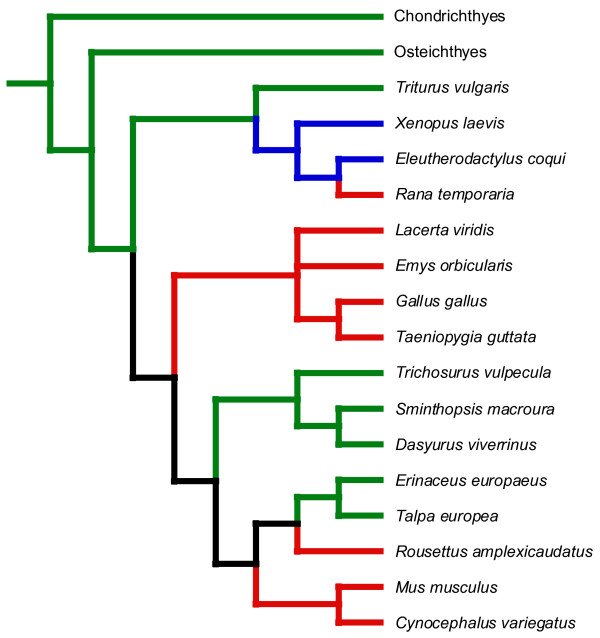
Reconstruction of the relative timing of forelimb and hindlimb development in tetrapods. Branches in blue and green show cases of significant hindlimb versus forelimb advances, respectively. Branches in red indicate synchrony and branches in black are equivocal according to the parsimony reconstruction. The tree is derived from references 33 and 34.

The relative timing of fore- and hindlimb development is labile in evolution, but with the observed pattern of change in timing showing a strong phylogenetic component. Different clades (anurans, diapsids, eutherians and marsupials) each have evolved characteristic timing relationships (see also Figure 2) that show no apparent correlation to the diverse lifestyles and adult morphologies of the species within each clade. The relative conservation of timing relationships within clades in the face of disparate adaptive needs is particularly striking among eutherians, which included a flying species (*Rousettus*), a glider (*Cynocephalus*), and a terrestrial species (*Mus*). Although the eulipotyphlan insectivores *Erinaceus *and *Talpa *do show a significant forelimb advance within and with respect to other eutherian mammals, this pattern is also consistent for the clade despite the different locomotory modes of the exemplars (terrestrial and fossorial, respectively). In short, the strong selection for modification of the adult limb morphology in eutherians did not produce correlated changes in the relative timing of forelimb-hindlimb development.

The only exceptions to the general pattern of clade-dependent conservation across tetrapods that we are aware of occur in lissamphibians. In anurans, the limbs develop approximately in synchrony in *Rana temporaria *[9] in contrast to the hindlimb acceleration found in the two species we examined. Among other lissamphibians, it is known that in some urodeles such as the smooth newt *Triturus vulgaris*, the forelimb is so accelerated relative to the hindlimb that it is chondrified before the bud of the latter has even appeared [10]. Forelimb advanced over the hindlimb also characterizes the Iberian ribbed newt *Pleurodeles watl *[11]. In other urodeles, such as the Siberian newt *Salamandrella keyserlingii *[12] and the clawed salamander *Onychodactylus japonica *[13], the forelimb is only marginally advanced if at all or, as in the Mexican plethodontid *Chiropterotriton magnipes*, the two limbs reportedly develop more or less simultaneously [14]. Increased species sampling may reveal yet additional variation within Lissamphibia.

Altogether, there was little evidence that adaptations of the adult tetrapod limb were associated with fore-/hindlimb sequence heterochrony during chondrogenesis. Instead, it appears that post-chondrogenic differences in growth rate (i.e., allometric heterochrony) play a more deciding role in shaping the final adult morphology of the tetrapod limb. A cogent example are marsupials, which often display a much larger hindlimb in adults despite the extreme acceleration of forelimb development in embryos. Similarly, the bat actually displayed the least degree of forelimb acceleration among the eutherians studied, despite the forelimbs being greatly enlarged and modified as wings in the adult. This morphology appears to derive from allometric heterochrony instead: the initially conserved pattern of chondrogenesis is followed by accelerated chondrocyte proliferation and differentiation, with increased *Bmp2 *expression and Bmp signalling [15]. A similar phenomenon of allometric and not sequence heterochrony was hypothesized by Jeffery et al. [16] to account for eye development in the spectral tarsier (*Tarsius spectrum*), a nocturnal primate with the largest relative eye-size among mammals.

An important question, therefore, is what is driving the changes in the early pattern of chondrogenesis among the major tetrapod clades, especially in light of the apparent conservation of this trait within most of them. Similar developmental pathways are responsible for the patterning and growth of fore- and hindlimbs, and covariation between both has been recorded statistically at different levels of the phylogenetic hierarchy in adults [17]. Yet, our study shows that heterochronic changes and dissociation have still occurred across major tetrapod clades and within lissamphibians to occasionally override the common mechanisms underlying limb development.

One possible explanation for the inferred transformations is that the different patterns are directly adaptive for the embryo. This has been hypothesized for marsupials and monotremes, where the greatly accelerated forelimb development has been linked to the necessity for the embryo to climb to the mother's marsupium to attach to a teat for further development [5]. Similarly, marsupials show another one of the few, clear instances of sequence heterochrony in mammals, with many cranial structures associated with feeding in the pouch also showing advanced development [5,18,19]. Similar adaptive explanations are lacking for the remaining species, however. This is particularly true for the two anurans examined here (*Eleutherodactylus*, *Xenopus*), which show a conserved pattern despite their very different developmental modes (direct versus indirect, respectively). Altogether, adaptive explanations seem difficult to postulate in general and for the amniotes in particular, the embryos of which develop in the protected environment of the cleidoic egg and so might be evolutionarily 'privileged' (sensu [20]) and shielded from diversifying selection.

An alternative, but not mutually exclusive, explanation is the presence of developmental 'constraints' [21] or that the overall pattern is the 'by-product' of other developmental processes. Although our findings are consistent with the existence of such constraints, they in no way can be taken as evidence of their existence.

We would add that the general pattern of fore- and hindlimb developmental timing we present based on 10 developmental landmarks belies its actual complexity. In *Dasyurus*, for example, although the forelimb buds appear before those of the hindlimb, a clear hindlimb bud is present throughout most of forelimb development. However, chondrogenesis does not start in the hindlimb buds until forelimb development is nearly complete. The initial timing difference between the appearance of fore- and hindlimb buds correlates with the strong craniocaudal developmental gradient observed at early stages of development [22]. However, the failure of the hindlimb bud to progress after its initial formation (a period of developmental 'dormancy'), is likely to be caused by more local factors, effectively maintaining a viable limb-bud whilst temporarily suspending outgrowth.

## Conclusion

We find that the relative timing of forelimb versus hindlimb development varies widely between major vertebrate clades. However, such forelimb-hindlimb sequence heterochronies are largely absent within major clades, the Lissamphibia forming a notable exception. The sequence differences that we did note between fore- and hindlimbs were not easy to explain in terms of morphological differences in the adult fore- and hindlimbs. Rather, the latter were more likely explained by allometric growth differences. The apparently conservative nature of forelimb versus hindlimb timing may mean that of skeletal chondrogenesis sequences could provide useful phylogenetic characters at higher taxonomic levels in tetrapods.

Our data, and those summarized in Rabl [23] and Richardson [24] allow an initial attempt to reconstruct the evolution of forelimb-hindlimb heterochrony across tetrapods (Figure 2). The primitive tetrapod condition appears to be for the forelimb to be advanced over the hindlimb (see also [25]). This condition characterizes the primitive condition for the homologous structures (the pectoral and pelvic fins, although many tetrapod autopodial structures are most likely absent in fishes [26]) in Chondrichthyes and Osteichthyes [24]. Thereafter, the evolution of this trait is equivocal (as indicated by the black branches in Figure 2), with two equally parsimonious solutions. One solution is for the forelimb advance to be retained ancestrally throughout tetrapods, with the individual groups (anurans, diapsids, marsupials, *Rousettus*, and *Mus *+ *Cynocephalus*) independently deviating from this pattern. The other solution is for a hindlimb advance to be a shared derived feature (synapomorphy) of amniotes, with marsupials and eulipotyphlan insectivores independently regaining the primitive tetrapod hindlimb advance.

Additional data will help clarify this picture, both by testing the reality of the apparent clade-specificity of forelimb-hindlimb heterochrony and by hopefully resolving outstanding regions of uncertainty. Key among the latter is within urodeles, where there is evidence that the pattern we present might not hold in unsampled species [10-14].

## Methods

We analysed developmental sequences in 138 embryos of 14 species, spanning a diverse taxonomic, ecomorphological and life-history breadth. Specimens were obtained from the Zeilstra and M.K. Richardson collections, Institute of Biology, University of Leiden; Hubrecht Laboratory, Netherlands Institute for Developmental Biology and Naturalis Museum, Leiden. We used whole-mounts prepared using standard protocols and viewed through an orange (G) filter to improve the contrast. Some histological sections were made to ensure that differences in staining time did not affect the scoring of skeletal elements. All had been collected in accordance with local ethical rules. Work on *Eleutherodactylus *was approved by the Harvard University, Faculty of Arts & Sciences, Standing Committee on the use of Animals in Research and Teaching, animal experimentation protocol (AEP) 99-09.

A total of 20 events was scored (Table 2), providing landmarks of development from the early bud stage to late chondrogenic events. All specimens were scored by JEJ. As a control, MKR independently scored a subset of several species; results were identical. The score '0' was assigned if an event had not yet occurred, and '2' if it had occurred. The score '1' was used to indicate an intermediate phase, such as if a cartilage element was stained but lacked distinct boundaries (i.e., it was a diffuse patch that graded imperceptibly into the surrounding tissue). We assumed the homology of the events, both amongst species (primary homology), and between the fore- and hindlimbs of individuals (serial homology).

**Table 2:** Limb developmental events scored in this analysis

***Forelimb event***	***Hindlimb event***
*External Form*	

A. bud first distinct	K. bud first distinct
B. AER appears	L. AER appears
C. digital plate crenation (fossae separating digits dorsally)	M. digital plate crenation (fossae separating digits dorsally)

*Chondrogenesis*	

D. humerus appears	N. femur appears
E. ulna appears	O. fibula appears
F. proximal carpal (ulnare) appears	P. proximal tarsal (fibulare) appears
G. carpal distal to ulnare appears	Q. tarsal distal to fibulare appears
H. metacarpal appears	R. metatarsal appears
I. proximal phalanx appears	S. proximal phalanx appears
J. distal phalanx appears	T. distal phalanx appears

Elements in the same horizontal row in the hand and foot are considered serially homologous for the purposes of this study.

Stylopodal and zeugopodal elements have clear homologues between the fore and hindlimbs (humerus/femur, radius/tibia and ulna/fibula). A basipodial element (ulnare/fibulare) also could be identified in most species. The total number of phalanges on a particular digit, however, varies amongst species. We therefore chose to record only the most proximal and distal phalanges, which provide proximal and distal landmarks for chondrogenesis in a given digit. The AER is absent in one of the anuran species considered here (*Eleutherodactylus coqui*; [7]), and very reduced in the other (*Xenopus laevis*; [27]).

Autopodials of the species studied vary in the number and pattern of prechondrogenic primordia, the pattern of fusion or regression during development, and in adult morphology (e.g., the numbers of digits and phalanges). We resolved these difficulties by looking at common (possibly plesiomorphic) patterns of development. In the forelimb, the digit adjacent to the ulna and ulnare is the first to develop [28,29]. We therefore selected this digit (and its associated metacarpal) as a proxy for overall digital development. A similar pattern is seen in the hindlimb with respect to the fibula and fibulare. In pentadactyl amniotes this digit invariably is digit IV.

### Reconstructing the developmental sequence

The embryonic age was unknown for most specimens. Even so, the very existence of intraspecific heterochrony means that absolute age usually cannot be considered to be a reliable criterion for ordering specimens in a developmental sequence [30]. We therefore used the parsimony-based 'Ontogenetic Sequence Analysis' (OSA [31]) on the limb character distribution amongst the specimens to find the most parsimonious developmental sequence for each species. OSA provides a rigorous framework for optimizing variations (polymorphisms) in the developmental sequence of a species to yield a single, consensus sequence. It relies on two assumed characteristics of development: (1) progressiveness – for any set of events, it is possible to conceive an early embryo in which none of the events has occurred, and a late embryo in which all the events have occurred; and (2) irreversibility – the same event cannot occur twice to the same organ.

Data for each species were analysed using the *TAXEQ2 *program [32], to identify specimens having actual or potentially equivalent scores (when missing data are taken into account). Such embryos were excluded from the analysis to reduce the computational time for OSA without the loss of any novel information.

### Intraspecific developmental rates

Comparisons of the relative rates of development between fore- and hindlimbs within each species were made using the event-pair method. This method encodes the relative timing between any two developmental events, with 0 indicating that event A occurred before event B, 1 indicating that both occurred simultaneously, and 2 indicating that event B occurred first. To highlight differential rates of development between the limbs without regard to any sequence changes within each limb we compared the 100 non-redundant event-pairs involving each homologous forelimb and hindlimb event (for a data set of *N *serially homologous events = *N*^2^; *N *= 10 here). All event pairs were scored from the standard 'perspective' of hindlimb-event vs. forelimb-event (i.e., rather than forelimb event vs. hindlimb event; cf. [18]).

The average event-pair score (EPS; i.e. the sum of all event-pair scores divided by the number of event-pairs) was analyzed to describe the overall timing of fore- and hindlimb development in each species. If fore- and hindlimbs developed synchronously, there would be 10 'simultaneous' event-pair scores (one for every pair of serially homologous events, each scoring 1) and 45 scores each of 'before' (scoring 0) and 'after' (scoring 2). Thus, the average value of EPS would be (10 × 1 + 45 × 0 + 45 × 2)/100 = 1. If the forelimb moved late relative to the hindlimb, the ratio of 'before' to 'after' scores would decrease, moving the average EPScloser to zero. Conversely, if the forelimb moved early relative to the hindlimb, the ratio of 'before' to 'after' scores would increase, moving the average EPS closer to two. The average EPS therefore provides an indicator of the relative timing of fore- and hindlimb development.

It is not clear precisely how the average EPS is affected by lack of resolution in a developmental sequence, but, in general, an increased number of simultaneous scores will tend to push the value towards one. Any lack of resolution is more likely to affect comparisons of events within the forelimb or the hindlimb, rather than the homologous events between the limbs, especially when the two sets of limbs develop asynchronously.

## Competing interests

The author(s) declares that there are no competing interests.

## Authors' contributions

JEJ and MKR collected the data, JEJ, ORPB-E and MKR carried out the analyses, MRS-V, ORPB-E, JH and MKR wrote the manuscript, MC advised on the analyses, JH, CP, LS, CtC, AR and CKO provided embryonic materials for the study and participated in discussions, and MKR conceived of the project. All authors read and approved the final manuscript.

## References

[B1] Huxley JS (1932). Problems of relative growth.

[B2] Polly PD, Hall BK (2007). Limbs in mammalian evolution. Fins into Limbs: Evolution, Development and Transformation.

[B3] Shubin N, Tabin C, Carroll S (1997). Fossils, genes and the evolution of animal limbs. Nature.

[B4] Gould SJ (1977). Ontogeny and Phylogeny.

[B5] Smith KK (2001). Heterochrony revisited: the evolution of developmental sequences. Biol J Linn Soc.

[B6] Nieuwkoop PD, Faber J (1967). Normal Table of Xenopus laevis.

[B7] Richardson MK, Carl T, Hanken J, Elinson RP, Cope C, Bagley P (1998). Limb development and evolution in *Eleutherodactylus coqui*, a frog with no apical ectodermal ridge (AER). J Anat.

[B8] Bellairs R, Osmond M (1998). The Atlas of Chick Development.

[B9] Kopsch FR (1952). Die Entwicklung des braunen Grasfrosches Rana fusca Roesel.

[B10] Duellman WE, Trueb L (1994). Biology of Amphibians.

[B11] Gallien L, Durocher M (1957). Table chronologique du développement chez *Pleurodeles waltlii *Michah. Bull Biol France Belgique.

[B12] Sitina LA, Medvedeva IM, Godina PV (1987). Development of *Hynobius keyserlingii *(in Russian). Akademia Nauka USSR, Nauka, Moscow.

[B13] Kuzmin SL (1995). The clawed salamanders of Asia.

[B14] Wake DB, Hanken J (1996). Direct development in lungless salamanders: what are the consequences for developmental biology, evolution and phylogenesis?. Int J Dev Biol.

[B15] Sears KE, Behringer RR, Rasweiler IV JJ, Niswander LA (2006). Development of bat flight: Morphologic and molecular evolution of bat wing digits. PNAS.

[B16] Jeffery JE, Bininda-Emonds ORP, Coates MI, Richardson MK (2002). Analysing evolutionary patterns in vertebrate embryonic development. Evol Dev.

[B17] Young NM, Hallgrimsson B (2005). Serial homology and the evolution of mammalian limb covariation structure. Evolution.

[B18] Jeffery JE, Richardson MK, Coates MI, Bininda-Emonds ORP (2002). Analyzing developmental sequences within a phylogenetic framework. Syst Bio.

[B19] Bininda-Emonds ORP, Jeffery JE, Richardson MK (2003). Is sequence heterochrony an important evolutionary mechanism in mammals?. J Mamm Evol.

[B20] Wolpert L (1994). The evolutionary origin of development: Cycles, patterning, privilege and continuity. Development Suppl.

[B21] Richardson MK, Chipman AD (2003). Developmental constraints in a comparative framework: A test case using variations in phalanx number during amniote evolution. J Exp Zool Mol Devel Evol.

[B22] Nelson JE (1992). Developmental staging in a marsupial *Dasyurus hallucatus*. Anat Embryol.

[B23] Rabl C (1910). Bausteine zu einer Theorie der Extramitäten der Wirbeltiere.

[B24] Richardson MK (1995). Heterochrony and the phylotypic period. Dev Biol.

[B25] Cole NJ, Tanaka M, Prescott A, Tickle C (2003). Expression of limb initiation genes and clues to the morphological diversification of threespine stickleback. Curr Biol.

[B26] Coates MI, Jeffery JE, Ruta M (2002). Fins to limbs: what the fossils say. Evol Dev.

[B27] Tarin D, Sturdee AP (1971). Early limb development of Xenopus laevis. J Embryol Exp Morphol.

[B28] Burke AC, Feduccia A (1997). Developmental patterns and the identification of homologies in the avian hand. Science.

[B29] Shubin NH, Alberch P, Hecht MK, Wallace B, Prance GI (1986). A morphogenetic approach to the origin and basic organization of the tetrapod limb. Evolutionary Biology.

[B30] Bininda-Emonds ORP, Jeffery JE, Coates MI, Richardson MK (2002). From Haeckel to event-pairing: the evolution of developmental sequences. Theory Biosci.

[B31] Colbert MW, Rowe T (2001). Ontogenetic sequence analysis: Using parsimony to characterize developmental hierarchies. J Morphol.

[B32] Wilkinson M (1995). TAXEQ2: software and documentation, v2.

[B33] Bininda-Emonds ORP, Cardillo M, Jones KE, MacPhee RDE, Beck RMD, Grenyer R, Price SA, Vos RA, Gittleman JL, Purvis A (2007). The delayed rise of present-day mammals. Nature.

[B34] Maddison DR, Schulz K-S, (eds) (1996). The Tree of Life Web Project. http://tolweb.org.

